# Adverse Childhood Events and Health Biomarkers: A Systematic Review

**DOI:** 10.3389/fpubh.2021.649825

**Published:** 2021-08-19

**Authors:** Sara Soares, Vânia Rocha, Michelle Kelly-Irving, Silvia Stringhini, Sílvia Fraga

**Affiliations:** ^1^EPIUnit - Instituto de Saúde Pública da Universidade do Porto, Porto, Portugal; ^2^Departamento de Ciências da Saúde Pública e Forenses e Educação Médica, Faculdade de Medicina, Universidade do Porto, Porto, Portugal; ^3^Faculty of Medicine Purpan, LEASP UMR 1027, Inserm-Université Toulouse III Paul Sabatier, Toulouse, France; ^4^Department of Epidemiology and Health Systems, Center for Primary Care and Public Health (Unisanté), University of Lausanne, Lausanne, Switzerland; ^5^Unit of Population Epidemiology, Division of Primary Care Medicine, Department of Community Medicine, Primary Care and Emergency Medicine, Geneva University Hospitals, Geneva, Switzerland

**Keywords:** biomarkers, biology of social adversity, ACES, review—systematic, adverse childhood events

## Abstract

**Background:** This systematic review aimed to summarize evidence reporting epigenetic and/or neuro-immuno-endocrine embedding of adverse childhood events (ACEs) in children, with a particular focus on the short-term biological effect of those experiences.

**Methods:** A search was conducted in PsycINFO®, PubMed®, Isi Web of Knowledge and Scopus, until July 2019, to identify papers reporting the short-term biological effects of exposure to ACEs.

**Results:** The search identified 58 studies, that were included in the review. Regarding exposure, the type of ACE more frequently reported was sexual abuse (*n* = 26), followed by life stressors (*n* = 20) and physical abuse (*n* = 19). The majority (*n* = 17) of studies showed a positive association between ACEs and biomarkers of the immune system. Regarding DNA methylation 18 studies showed more methylation in participants exposed to ACEs. Two studies presented the effect of ACEs on telomere length and showed that exposure was associated with shorter telomere length.

**Conclusion:** Overall the associations observed across studies followed the hypothesis that ACEs are associated with biological risk already at early ages. This is supporting evidence that ACEs appear to get “under the skin” and induce physiological changes and these alterations might be strongly associated with later development of disease.

## Introduction

Adverse childhood experiences (ACEs) are stressful and traumatic events that occur in childhood and adolescence, until the age of 18 years and encompass various aspects of family dysfunction such as experiences of sexual abuse, physical or emotional abuse, and physical neglect (1). These experiences cause suffering to children (2) and undermine their sense of safety, stability, and bonding (3), and consequently impact their normal growth and development (4).

ACEs have been compellingly associated with a life-long increased risk for psychopathology and stress-related chronic health problems (5–10). Evidence shows that exposure to ACEs is strongly associated with a higher likelihood of developing ischemic heart disease, cancer, stroke, chronic bronchitis, emphysema or diabetes later in life and even with pre-mature death (1, 2, 11, 12). However, the potential mechanisms involved in the biological embodiment of social adversity in early ages that would be translated into an increased risk of disease later in life are still not fully understood (13–15).

Two main biological pathways are proposed to explain how the ACEs “get under the skin” and be associated with later negative health outcomes. Indirectly, it can be explained by the adoption of unhealthy behaviors (e.g., poor diet, sedentary behavior, smoking), that are socially patterned and thus more likely to be acquired by individuals from contexts of greater social adversity, and also associated with increased risk of disease later in life; or *via* a direct physiological disruption of regulatory pathways responsive to stress caused by adverse experiences. These alterations might be precursors of disease onset later in life, may start to operate early in life and be tracked over the life course. Exposure to adverse experiences may result in a variety of physiological changes during childhood (2, 16), including epigenetic mechanisms (13, 15), alteration of neural function and structure (13–15), increased activation of neurobiological systems, such as the hypothalamic-pituitary-adrenal (HPA) axis or the sympathetic nervous system (16, 17). Therefore, increased activation of these systems leads to a cascade of physiological processes (16–18), which in adults, was linked with the development of central fat, dysregulated carbohydrate metabolism and the accumulation of blood lipids in the arterial lining, all of which accelerate chronic disease development (19).

Evidence allows us to hypothesize that exposure to adversity during the first years of life might already be biologically embedded well before adult life, independently of the effects of behaviors in this association. Exposure to stressful circumstances between conception into adolescence causes a cascade of physiological responses that may modify an individual's biology in the long term in a way that makes them vulnerable to develop disease later in life (7, 9, 18, 20).

As a biomarker or a biological marker is a measurable indicator of some biological state or condition and is often measured and evaluated to examine normal biological processes, pathogenic processes, or pharmacologic responses to therapeutic intervention, in this work we aimed to identify biomarkers that are part of biological/physiological systems and therefore can suffer alterations as a result of exposure to adversity. We know that ACEs impact a child's life, and those “scars” can be identified and are perceptible, such as internalizing (e.g., anxiety, depression) and externalizing (e.g., aggression) problems and learning difficulties (21). This review aims to investigate the “hidden” effects of such exposures on children's biology that can be measured and quantified and may have a major impact already in childhood but can also have the potential to be programming children's health and translating into negative health outcomes later in life.

Thus, identify the physiological systems that may be immediately affected by the exposure to adversity already at early ages would allow understanding the pathways by which ACEs may impact later development of disease, to estimate the impact of ACEs would have later in life, and consequently define interventions to protect children in a trajectory of increased risk of poor health or to mitigate the effects already in place to avoid the development of disease in the adult life. Therefore, this review aims to systematically summarize evidence reporting epigenetic and/or neuro-immuno-endocrine embedding of adverse experiences in childhood. Specifically, it aims to describe which ACEs have been associated within a short time span until quantification of biological markers, to identify which physiological systems have been more investigated to explain the association between ACEs and later development of disease, and finally, to describe the impact and consequences of ACEs on the normal functioning of physiological systems. In addition, it is intended to discuss potential methodological issues that might explain inconsistencies among studies, which should be addressed and enhanced in future research.

## Methods

### Search Strategy

PsycINFO®, PubMed®, Isi Web of Knowledge and Scopus were searched until July 2019, to identify published papers reporting biological effects of exposure to ACEs before the age of 18 years. The keywords were chosen based on the literature and previously published theoretical reviews (22) and systematic reviews (23, 24), according to the usually used markers to measure biological alterations, adapted to each database and included the following terms: child maltreatment, child trauma, child adversity, early life stress, child abuse, child neglect, emotional stress, violence, bullying, and C-reactive Protein, CRP, Tumor Necrosis Factor, TNF-α, cytokine, interleukin, IL-6, inflammatory, inflammation, fibrinogen, white blood cell, methylation, DNA, DNA methylation, nervous system, amygdala, amygdala volume, hippocampus, hippocampal volume, prefrontal cortex volume, endocrine system, HPA axis, cortisol.

### Selection of Studies

**Figure 1:**
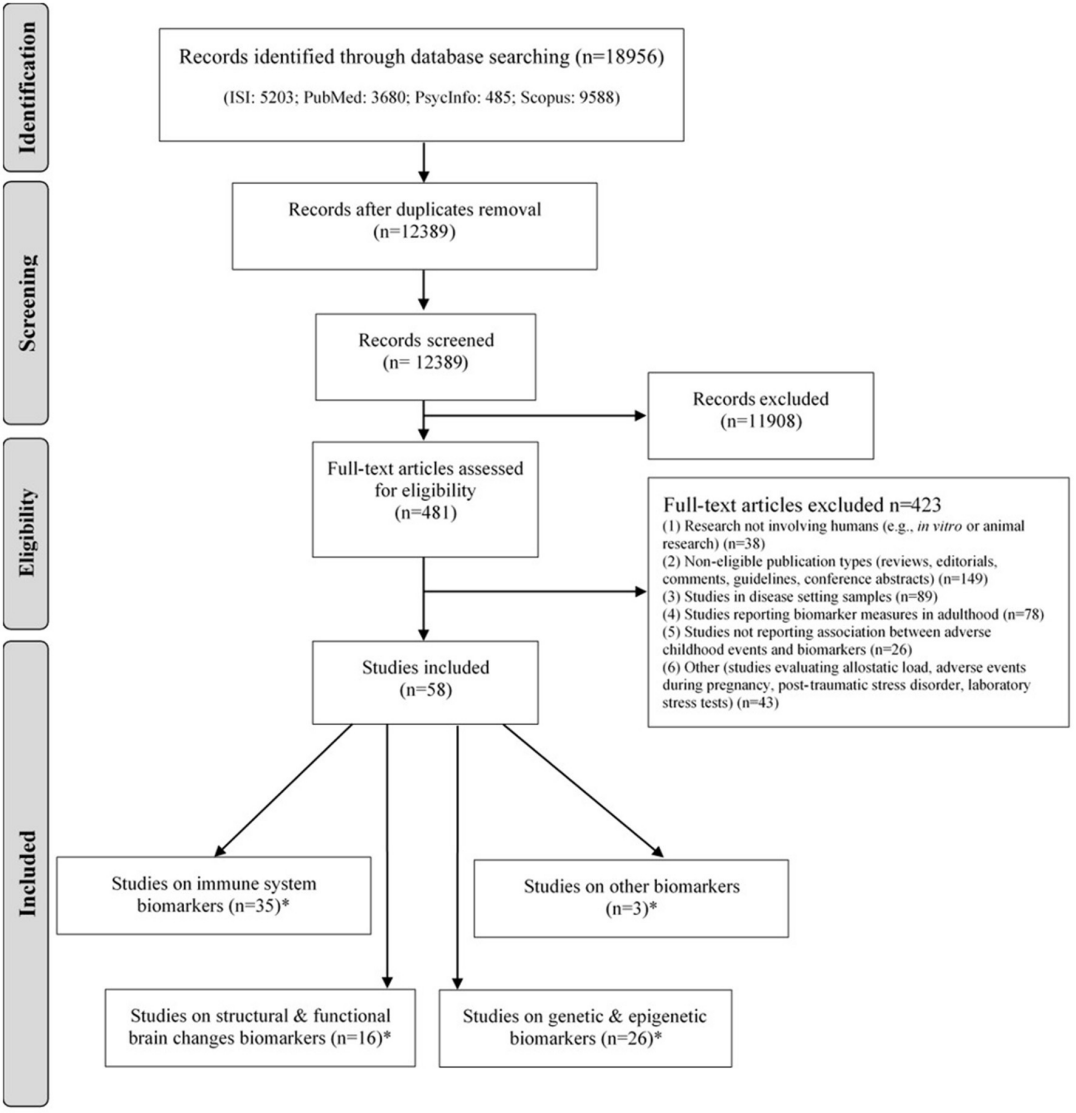
PRISMA flow diagram of the literature search.

The list of references retrieved was screened independently by two reviewers (SSo and VR), following pre-defined criteria, to determine the eligibility of each article (Figure 1). Inclusion criteria are as following: case-control and cohort studies; original research; studies evaluating adverse childhood experiences; studies reporting biomarker measures in adulthood (≤18 years old); studies reporting an association between ACEs and biomarkers. The criteria for exclusion of studies were the following: (1) research not involving humans (e.g., *in vitro* or animal research); (2) non-eligible publication types (reviews, editorials, comments, guidelines, conference abstracts); (3) studies in disease setting samples; (4) studies reporting biomarker measures in adulthood (>18 years-old); (5) studies not reporting an association between ACEs and biomarkers; (6) other (studies evaluating allostatic load, adverse experiences during pregnancy, post-traumatic stress disorder, laboratory procedures to induce stress).

ACEs were defined considering Felitti exposure categories (1), namely psychological, physical and sexual abuse, and household dysfunction. Also, we included in the review any adverse experiences involving close relationships (caregivers, family and peers). Then, adverse experiences were categorized into: sexual abuse (includes any type of sexual abuse reported), life stressors (that includes a more thorough and comprehensive summary of adversities related with relationships such as the death of a family member, trouble with a teacher, exposure to community violence), physical abuse (includes abuse perpetrated by parents, caregivers or other relatives and by teachers) and physical neglect (includes physical neglect by parents or other caregivers). Biomarkers were defined according to the definition from the International Program on Chemical Safety, led by the World Health Organization (WHO) and in coordination with the United Nations and the International Labor Organization, as “*any substance, structure, or process that can be measured in the body or its products and influence or predict the incidence of outcome or disease*” (25). Biological markers were then divided by the biological mechanism with which they fitted better (Table 1).

**Table 1:** Description of biological markers divided by the biological mechanism.

**Biological marker**	**Description**
**Immune system**	
CRP	Acute-phase protein of hepatic origin whose circulating concentrations rise in response to inflammation.
IL-6	Important mediator of fever and of the acute phase response
TNF-α	Cytokine involved in systemic inflammation and one of the cytokines that make up the acute phase reaction.
IL-1b	Cytokine and important mediator of the inflammatory response, involved in a variety of cellular activities, including cell proliferation, differentiation, and apoptosis.
IL-10	Cytokine with multiple, pleiotropic, effects in immunoregulation and inflammation.
IL-12p70	Interleukin naturally produced by dendritic cells, macrophages and neutrophils, that stimulates the production of interferon-gamma and TNF-α from T cells and natural killer cells.
IL-8	Induces chemotaxis in target cells, primarily neutrophils but also other granulocytes, causing them to migrate toward the site of infection, also stimulates phagocytosis once they have arrived.
Cortisol	Prevents the release of substances in the body that cause inflammation).
**Structural and functional brain changes**	
BDNF	Acts on certain neurons of the central nervous system and the peripheral nervous system, helping to support survival of existing neurons, and encouraging growth and differentiation of new neurons and synapses.
Hippocampal volume	Chronic stress resulting in elevated levels of cortisol, is seen to be a cause of neuronal atrophy in the hippocampus; this atrophy results in a smaller hippocampal volume.
Amygdala volume and amygdala functional connectivity	Amygdala is a key region of the brain and plays a crucial role in processing fear, mediates the ability to associate emotional significance to a formerly neutral stimulus, triggers a host of adaptive responses to threatening stimuli, for example, by regulating the magnitude and duration of serotonergic responses.
Gray matter	Contains most of the brain's neuronal cell bodies; includes regions of the brain involved in muscle control, and sensory perception such as seeing and hearing, memory, emotions, speech, decision making, and self-control.
Neurologic abnormalities	Structural, biochemical or electrical abnormalities in the brain, spinal cord or other nerves.
Pituitary gland volume	Mediates the stress response, *via* the hypothalamic–pituitary–adrenal axis and can be adversely affected by an over- or under-production of associated hormones.
Voxel-based morphometry	Technic that allows the detection of focal microstructural differences in brain anatomy *in vivo* between groups of individuals without requiring any *a priori* decision concerning which structure to evaluate.
**Genetic and epigenetic**	
Methylation	The addition of a methyl group on a substrate, or the substitution of an atom (or group) by a methyl group. DNA methylation, including how it occurs and where it occurs, is an important component in numerous cellular processes, including embryonic development, genomic imprinting, X-chromosome inactivation, and preservation of chromosome stability. Given the many processes in which methylation plays a part, errors in methylation to a variety of harmful consequences, including several human diseases.
Telomere length	Telomeres, the specific DNA–protein structures found at both ends of each chromosome, protect genome from nucleolytic degradation, unnecessary recombination, repair, and inter-chromosomal fusion. Telomeres therefore play a vital role in preserving the information in genome. As a normal cellular process, a small portion of telomeric DNA is lost with each cell division, and telomere length reaches a critical limit, the cell undergoes senescence and/or apoptosis. Thus, telomere length may serve as a biological clock to determine the lifespan of a cell and an organism.
Copeptin	Copeptin measurement has been useful in various clinical indications, including the diagnosis of diabetes insipidus and the monitoring of sepsis and cardiovascular diseases, particularly, closely linked to the pathophysiological pathways of heart failure and acute coronary syndrome.
Leptin	The roles of leptin include regulation of energy homeostasis, neuroendocrine function, metabolism and regulation of immune function. Circulating leptin levels serve as an indicator for energy reserves and directs the central nervous system to adjust food intake and energy expenditure accordingly. Leptin exerts immediate effects by acting on the brain to regulate appetite.
Dehydroepiandrosterone (DHEA)	DHEA is reported to reduce proliferation of human aortic smooth muscle cells, and to improve cellular immune function, after inhibiting apoptosis. Furthermore, DHEA may have beneficial effect in patients with atherosclerosis, immunodeficiency disease or inflammatory disease.

The decisions taken independently by the authors in each step were compared, and discrepancies were solved by consensus or after discussion with a third researcher (SF). PRISMA flow diagram of the literature search is depicted in Figure 1.

### Data Extraction

Two investigators (SSo and VR) independently extracted data from 58 studies regarding the year of publication, country, and region where the study was conducted, sample characteristics (sample, sample size, participant's age, female proportion, type of ACEs, the instrument used to measure adverse experiences, age at event exposure and biological marker assessed).

### Data Synthesis and Analysis

Two summary tables of results were created, compiling the extracted information (Tables 2, 3). Studies were divided according to the different development phases of growth using the age at which ACEs occurred, as following: toddlerhood (0–2 years); childhood (3–12 years and further classification into play from 3 to 5 years and middle childhood from 6 to 12 years); and adolescence (13–18 years and divided in mid-adolescence from 13 to 15 years and late adolescence from 15 to 18 years). Due to heterogeneity of ACEs measures, analytic methods and in the biomarkers, a qualitative description of the association and the strength of the reported association were assigned based on the magnitude of the reported effect measures (85), defined according to the author's results description, as strong or weak, and statistical significance of the provided results. Results were then summarized in a table presenting positive and inverse associations (associations were classified as positive when authors reported that participants exposed to adverse experiences presented higher levels of biological markers, and as inverse when a decrease in the biological markers when adversity was reported), and the strength of association (Table 4).

**Table 2:** Descriptive characteristics of all included studies (*n* = 58).

**References**	**Sample size**	**Participants' age (years) (range/mean)**	**Female proportion (%)**	**Type of ACE**	**Instrument to assess ACE**	**Age at the ACE (years)**	**Age at the measure of biomarker**	**Time between exposure and biomarker measure^a^**	**Biomarker**	**Quality^b^**
**Toddlerhood: 0–3 years**										
Bhopal et al. (26)	T: 436	12.4	n.m.	Life stressors^*^	n.m.	12 months	1	0–1	Cortisol	22
Dahmen et al. (27)	T: 51	ACE+: 10.6	ACE+:50.0	Maltreatment	German self-report questionnaire	0–3	10.6	7.6–10.6	Hippocampal volume	19
	ACE+: 25	ACE-: 10.4	ACE-: 44.0							
**Childhood: 3–12 years**										
Bucker et al. (28)	T: 62	ACE+:9.44	ACE+:38.9	Sexual abuse, maltreatment, and/or neglect	n.m.	3–12	3–12	0–9	IL-12p70, IL-6, IL-8, IL-10, IL1β, TNF-α and BDNF	19
	ACE+:36	ACE-: 8.96	ACE-: 42.3							
Chen et al. (29)	T: 516	ACE+: 10	ACE+:40.2	Life stressors^*^	Exposure to violence	Lifetime	0–9	0–9	DNA methylation (ADCYAP1R1)	21
	ACE+: 271	ACE-: 11	ACE-: 50.6		Scale questionnaire					
Cicchetti and Handley (30)	T: 534 ACE+: 285	T: 9.41 ACE+: 9.45 ACE-: 9.97	48.5	Abuse and neglect	Maltreatment classification system	Lifetime	9.4	9.4	DNA methylation (NR3C1)	21
Cicchetti et al. (31)	T: 489	8–12 (*M* = 9.72)	ACE+:42.7	Abuse and neglect	Maltreatment Classification System	0–9	0–9	0–9	CRP	21
	ACE+: 267		ACE-: 53.7							
Fujisawa et al. (32)	T: 85	*M* = 12.9	35.3	Physical, emotional, and sexual abuse, physical and emotional neglect	n.m.	Early in life	12.9	-	DNA methylation	19
	ACE+: 44									
Shalev et al. (33)	T: 236	T1: 5	49.2	Life stressors^*^, bullying and physical maltreatment	n.m.	5–10	5–10	0–5	Telomere length	21
		T2: 10								
Slopen et al. (34)	T: 5,802	IL-6: 10 and 15	49.8	Life stressors^*^ and sexual abuse	n.m.	0–8	10–15	0–15	IL-6; CRP	22
		CRP: 10								
**Play: 3–5 years**										
Bruce et al. (35)	T: 177	3–6	ACE+:46.0	Physical and sexual abuse, physical neglect, and emotional maltreatment	Maltreatment classification system	Lifetime	3–6	3–6	Cortisol	15
	ACE+: 117		ACE-:47.0							
Parade et al. (36)	T: 231	51.2 months	52.4	Physical and sexual abuse, physical neglect, and emotional maltreatment	System for coding subtype and severity of maltreatment in child protective records	3–5	3–5	0–2	DNA methylation	20
	ACE+: 123									
Parent et al. (37)	T: 260 ACE+: 134	3–5 ACE+: 8.1 ACE-: 8.4	53.8	Physical and sexual abuse, physical neglect and emotional maltreatment	The diagnostic infant and preschool assessment	Past 6 months	3–5	0.5	DNA methylation	21
Tyrka et al. (38)	T: 184	3–5	51.1	Physical and sexual abuse, physical neglect, and emotional maltreatment	Diagnostic infant and preschool assessment	Past 6 months	3–5	0.5	DNA methylation (NR3C1)	20
Tyrka et al. (39)	T: 174	3–5	51.7	Physical and sexual abuse, physical neglect, and emotional maltreatment	Diagnostic infant and preschool assessment	Past 6 months	3–5	0.5	DNA methylation (FKBP5 and NR3C1)	19
**Middle childhood (6–12 years)**										
Baldwin et al. (40)	T: 1,732	18.4	51.3	Several types of victimization	n.m.	5, 7, 10, 12	18	6–13	CRP	21
Bevans et al. (41)	T: 68	7.6–13.8 (*M* = 10.7)	56.0	Life stressors^*^	The life events checklist and UCLA PTSD index for DSM-IV child- and parent-report versions	Lifetime	10.7	10.7	Cortisol	14
Buchweitz et al. (42)	33	10–14 (*M* = 11.45)	42.4	Life stressors^*^ and sexual abuse	Juvenile victimization questionnaire (reduced version)	Lifetime	11.4	11.4	Cortisol	17
Bush et al. (43)	T: 178	9–11 (*M* = 10.92)	47.0	Life stressors^*^	n.m.	Lifetime	10.9	10.9	DNA methylation	21
Cicchetti et al. (44)	T: 548	*M* = 9.40	47.8	Abuse and neglect	Maltreatment classification system	Lifetime	9.4	9.4	DNA methylation	21
	ACE+: 298									
Cicchetti et al. (45)	T: 384	*M* = 9.25	39.5	Abuse and neglect	Maltreatment classification system	Lifetime	9.25	9.25	Cortisol	22
Coelho et al. (46)	T: 136	ACE+: 9.44	ACE+:47.8	Physical, emotional and sexual abuse, physical and emotional neglect	Childhood trauma questionnaire	Lifetime	9.4	9.4	Copeptin	20
	ACE+: 65	ACE-: 8.99	ACE-: 52.2							
Danese et al. (47)	T: 172	12	n.m.	Physical maltreatment	Childhood trauma questionnaire	5–12	12	0–7	Leptin and CRP	19
	ACE+: 81									
Doom et al. (48)	T: 341	*M* = 8.4	49.6	Physical, emotional and sexual abuse, physical and emotional neglect	Maltreatment classification system	Lifetime	8.4	8.4	Cortisol	18
	ACE+: 187									
Doom et al. (49)	T: 247	7.9–10.9 (*M* = 9.42)	47.8	Abuse and neglect	Maltreatment classification system	Lifetime	9.42	7.9–10.9	Cortisol and DHEA	18
	ACE+: 137									
Drury et al. (50)	T: 80 ACE+: 46	5-15 (*M* = 10.2) ACE+: *M* = 0.4 ACE-: *M* = 9.9	T: 49.0 ACE+:57.0 ACE-:38.0	Life stressors^*^	Part of preschool age psychiatric assessment	Lifetime	10.2	10.2	Telomere length	18
Huang et al. (51)	T: 32	ACE+= 16.0	ACE+:53.8	Physical and sexual abuse, and/or witnessed domestic violence	Childhood adversity interview	<10 (persistent for ≥6 months)	15.89	0–10	Voxel-based morphometry	21
	ACE+=19	ACE-: 15.9	ACE-:73.7							
Naumova et al. (52)	T: 28	7–10	32.1	Foster care	n.m.	Lifetime	8.14	8.14	DNA methylation	20
		ACE+: *M* = 8.14								
		ACE-: *M* = 8.35								
Non et al. (53)	T: 136	12.5	ACE+:48.0	Foster care	n.m.	Lifetime	12	12	DNA methylation	21
	ACE+: 82		ACE-:51.0							
Park et al. (54)	T: 79	4.0-8.0 (*M* = 6.1)	50.6	Life stressors^*^	Life events scale for young children	Past 12 months	6.06	1	Amygdala functional connectivity	20
Romens et al. (55)	T: 56	11–14 (*M* = 12.1)	46.4	Physical maltreatment	Child protective services records	Lifetime	12.1	12.1	DNA methylation (NR3C1)	20
	ACE+: 18									
Simsek et al. (56)	T: 76	ACE+: *M* = 13.4	ACE+: 28.0	Sexual abuse	n.m.	11.7	13.4	1.7	Cortisol	21
	ACE+: 38	ACE-: *M* = 13.5	ACE-: 28.0							
Stroud et al. (57)	T: 113	12.3	100	Life stressors^*^	Youth Life stress interview	Lifetime	12.3	12.3	Cortisol	21
Trickett et al. (58)	T: 173	6–16 (*M* = 11)	100	Sexual abuse	n.m.	7.8	6–16	6–16	Cortisol	21
	ACE+: 84									
Vaillancourt et al. (59)	T: 154	147 months	51.9	Bullying	Adapted from (60)	Past 3 months	12.2	0.25	Cortisol	19
Whittle et al. (61)	T: 117	12.7	48.7	Physical and sexual abuse, physical neglect, and emotional maltreatment	Childhood trauma questionnaire	<12	12.7	12.7	Hippocampal and amygdala volumes	21
Yang et al. (62)	T: 192	5–14 (*M* = 10.2)	58.0	Physical, sexual, emotional abuse and witnessed domestic violence	n.m.	Past 6 months	10.2	0.5	DNA methylation	21
	ACE+: 96									
**Adolescence: 13–18 years**										
Cicchetti et al. (63)	T: 60	ACE+: 9–15 (*M* = 11.31)	ACE+:60.0	Abuse	Maltreatment and abuse chronology of exposure (pediatric version)	Lifetime	9–15	9–15	DNA methylation	21
	ACE+: 35	ACE-: 10–14 (*M* = 11.76)	ACE-:56.0							
Cisler (64)	T: 56 ACE+: 26	11–17 ACE+: 15.2 ACE-: 14.7	100	Physical, emotional, and sexual abuse, physical and emotional neglect	National survey of adolescents and childhood trauma questionnaire	Lifetime	11–17	11–17	Amygdala functional connectivity	16
Copeland et al. (65)	T: 1,309	9–16	52.5	Bullying	Bullying part of CAPA	9–16	9–16	0–7	CRP	21
Humphreys et al. (66)	T: 178	9.1–14.0 (*M* = 11.4)	57.0	Life stressors^*^, physical and sexual abuse	Traumatic events screening inventory for children	Lifetime	9.1–14.0	9.1–14.0	Hippocampal volume	17
Ito et al. (67)	T: 104	13.0	49.0	Physical, emotional, and sexual abuse	Medical records and the department of social services records	Lifetime	13	13	Neurological abnormalities	15
Kaess et al. (68)	T: 69	12.62	30.0	Physical, emotional, and sexual abuse, physical and emotional neglect	Childhood trauma questionnaire	Lifetime	14–16	14–16	Pituitary gland volume	20
Malhi et al. (69)	T: 201	12–17	100	Emotional abuse and/or neglect	Childhood trauma questionnaire	Lifetime	12–17	12–17	Hippocampal volume	20
Östberg et al. (70)	T: 198	14–16	59.2	Bullying	Pressure and activation stress scale	Lifetime	14–16	14–16	Cortisol	19
Pagliaccio et al. (71)	T: 120	9–14 (*M* = 11.2)	48.3	Life stressors^*^	Preschool-age psychiatric assessment and childhood and adolescent psychiatric assessment	Lifetime	9–14	9–14	Amygdala functional connectivity	22
Ruttle et al. (72)	T: 330	14.5–19.2	n.m.	Life stressors^*^	Adolescent perceived events scale and the life events survey	9–18	14.5–19.2	1.2–10.2	Cortisol	20
Saxbe et al. (73)	T: 21	*M* = 16.9	43.0	Life stressors^*^	Survey of children's exposure to community violence, domestic conflict index and conflict tactics scale–parent/child	11.79–13.93	16.92	2.99–5.13	Amygdala and hippocampal volume	21
Simsek et al. (74)	T: 86 ACE+: 44	8–17 ACE+: 13.1 ACE-: 13.8	ACE+:72.7 ACE-:71.4	Sexual abuse	n.m.	22.72 months before examination	8–17	1.9	Cortisol, BDNF	18
**Mid adolescence: 13–15 years**										
Efstathopoulos et al. (75)	T: 1,149	13–14	54.4	Bullying and Life stressors^*^	n.m.	Lifetime	13–14	13–14	DNA rmethylation (NR3C1)	20
**Late adolescence: 15–18 years**										
Edmiston et al. (76)	T: 42	12–17 (*M* = 15.3)	50.0	Physical, emotional and sexual abuse, physical and emotional neglect	Childhood trauma questionnaire	Lifetime	15.33	15.33	Gray Matter	18
Esposito et al. (77)	T: 83	ACE+: 12.7–18.7 (*M* = 15.7)	ACE+:50.0 ACE-: 54.5	Life stressors^*^	The life events checklist (child/adolescent version)	Past year	15	1	DNA methylation	19
	ACE+:50	ACE-: 13.0–17.2 (*M* = 15.4)								
**0–18 years**										
Marzi et al. (78)	T: 1,468	18	n.m.	Domestic violence, bullying, physical maltreatment, sexual abuse, emotional abuse and neglect, and physical neglect	Juvenile victimization questionnaire and childhood trauma questionnaire	5, 7, 10, and 12 and 12–18	18	0–6	DNA methylation (NR3C1)	21
Radtke et al. (79)	T: 46	*M* = 15	60.9	Life stressors^*^, physical, emotional and sexual abuse, physical and emotional neglect	KERF-I	<18	11–18	0–18	DNA methylation (NR3C1)	19
Serbulent et al. (80)	T: 27	ACE+: 3–16 (*M* = 15)	74.0	Sexual abuse	n.m.	72 h before the examination	0–18	72 h	IL6, IL10, cortisol	22
	ACE+: 17	ACE-: 6–16 (*M* = 10.4)								
Tyborowska et al. (81)	T: 37	*M* = 14.6 and *M* = 17.1	22.0	Life stressors^*^	Life events questionnaire and Coddington's life events scale for children	<5 and 14–17	0–17	0–17	Gray matter volume	20
Van Der Knaap et al. (82)	T: 468	14–18 (*M* = 16.1)	50.4	Life stressors^*^	n.m.	0–15	16.1	1.1–16.1	DNA methylation	20
Van Der Knaap et al. (83)	T: 939	*M* = 16.2	n.m.	Life stressors^*^	Childhood trauma questionnaire (adaptation)	0–15	16.2	1.2–16.2	DNA methylation (SLC6A4)	22
White et al. (84)	T: 537	3–16 ACE+: *M* = 9.86 ACE-: *M* = 10.08	50.6 ACE+:46.1 ACE-:54.5	Physical and sexual abuse, physical neglect and emotional maltreatment	Maltreatment classification system	Lifetime	3–16	3–16	Cortisol	22

a *Time between exposure to ACEs and measure of biomarker*.

b *Quality of reporting of the included studies was assessed using the Strengthening Reporting of Observational Studies in Epidemiology (STROBE) Statement: Guidelines for Reporting Observational Studies. All studies scoring higher than the median in the STROBE checklist for cohort, case-control, and cross-sectional studies (combined) and thus revealing a satisfactory to good quality were included*.

* *Life stressors (e.g., death of a family member, trouble with a teacher)*.

**Table 3:** Descriptive characteristics of all included studies (*n* = 58).

**References**	**Country**	**Study design**	**Sample**	**Year of the survey**	**Prevalence of ACEs (%)**
**Toddlerhood: 0–3 years**					
Bhopal et al. (26)	India	Longitudinal	SPRING-ELS	2015	n.m.
Dahmen et al. (27)	Germany	Case-control	Community	2006–2007	Amongst cases: 51.0
**Childhood: 3–12 years**					
Bucker et al. (28)	Brazil	Case-control	Multi-cohort	n.m.	Amongst cases: Neglect: 91.75 Physical abuse: 52.8 Sexual abuse: 19.4
Chen et al. (29)	Puerto Rico	Case-control	Neighborhood clusters	2009–2010	1.20
Cicchetti and Handley (30)	USA	Case-control	Research summer camp program	n.m.	Amongst cases: Emotional maltreatment: 62.5 Neglect: 75.4 Physical abuse: 28.4 Sexual abuse: 8.8
Cicchetti et al. (31)	USA	Case-control	Research summer camp program	n.m.	Amongst cases: 54.6
Fujisawa et al. (32)	Japan	Case-control	Community	n.m.	Amongst cases: 52.4
Shalev et al. (33)	United Kingdom	Longitudinal	Environmental-risk study	1995 2000	Overall: 45.8 Bullying: 24.1 Domestic IPV: 16.9 Physical maltreatment: 26.7
Slopen et al. (34)	USA	Longitudinal	Avon longitudinal study of parents and children	n.m.	n.m.
**Play: 3–5 years**					
Bruce et al. (35)	USA	Case-control	Community	n.m.	Amongst cases: 68.8
Parade et al. (36)	USA	Case-control	Community	n.m.	53.0
Parent et al. (37)	USA	Longitudinal	Community	n.m.	51.5
Tyrka et al. (38)	USA	Cross-sectional	Community	n.m.	Amongst cases: Emotional maltreatment: 66.2 Lack of supervision: 27.0 Neglect: 12.2 Physical abuse: 12.2 Sexual abuse: 21.6
Tyrka et al. (39)	USA	Cross-sectional	Community	n.m.	Amongst cases: Emotional maltreatment: 68.1 Lack of supervision: 30.4 Neglect: 11.6 Physical abuse: 11.6 Sexual abuse: 18.8
**Middle childhood (6–12 years)**					
Baldwin et al. (40)	United Kingdom	Longitudinal	Environmental risk	1994–1996 to 2012–2014	26.5
			Longitudinal twin study		
Bevans et al. (41)	USA	Cross-sectional	Community	n.m.	n.m.
Buchweitz et al. (42)	Brazil	Cross-sectional	Community	n.m.	Lifetime: 82.5 Last year: 72.5
Bush et al. (43)	USA	Longitudinal	Peers and Wellness Study	2003–2005; 2010	n.m.
Cicchetti et al. (44)	USA	Case-control	Research summer camp program	n.m.	Amongst cases: Emotional abuse: 59.4 Neglect: 71.2 Physical abuse: 27.2 Sexual abuse: 8.7
Cicchetti et al. (45)	USA	Case-control	Research summer camp program	n.m.	Amongst cases: Emotional maltreatment: 74.3 Neglect: 79.4 Physical abuse: 37.1 Sexual abuse: 16.6
Coelho et al. (46)	Brazil	Cross-sectional	High Risk Cohort Study for Psychiatric Disorder	n.m.	Amongst cases: 47.8
Danese et al. (47)	USA	Case-control	Environmental-Risk Longitudinal Twin Study	n.m.	n.m.
Doom et al. (48)	USA	Case-control	Multi-cohort	n.m.	Amongst cases: Emotional maltreatment: 49.7 Neglect: 66.3 Physical abuse: 29.9 Sexual abuse: 6.4
Doom et al. (49)	USA	Case-control	Summer camp program	n.m.	Amongst cases: Emotional abuse: 49.7 Neglect: 66.3 Physical abuse: 29.9 Sexual abuse: 6.4
Drury et al. (50)	USA	Case-control	Community	n.m.	57.0
Huang et al. (51)	USA	Case-control	Part of a larger study	n.m.	14.7
Naumova et al. (52)	Russia	Case-control	Community	n.m.	Amongst cases: 50.0
Non et al. (53)	Romania	Case-control	Bucharest early intervention project	n.m.	Amongst cases: 50.0
Park et al. (54)	USA	Cross-sectional	Part of two larger studies	n.m.	n.m.
Romens et al. (55)	USA	Case-control	Community	n.m.	32.0
Simsek et al. (56)	Turkey	Case-control	Department of Child Psychiatry at Dicle University Hospital	May–November 2012	n.m.
Stroud et al. (57)	USA	Case-control	Part of a larger study	n.m.	n.m.
Trickett et al. (58)	USA	Case-control	Community	n.m.	n.m.
Vaillancourt et al. (59)	Canada	Cross-sectional	Community	n.m.	Physical bullying: 20.8 Social bullying: 43.5 Verbal bullying: 58.4
Whittle et al. (61)	Australia	Longitudinal	Orygen adolescent development study	n.m.	n.m.
Yang et al. (62)	USA	Case-control	Community	2011	Amongst cases: Emotional abuse: 65.0 Neglect: 83.0 Physical abuse: 65.0 Sexual abuse: 24.0 Witness domestic violence: 70.0
**Adolescence: 13–18 years**					
Cicchetti et al. (63)	Tanzania	Case-control	Community	n.m.	n.m.
Cisler (64)	USA	Case-control	Community	n.m.	Amongst cases: 46.4
Copeland et al. (65)	USA	Longitudinal	Great smoky mountains study	n.m.	n.m.
Humphreys et al. (66)	USA	Cross-sectional	Part of a larger study	n.m.	98.0 (at least 1 event > 6 years)
Ito et al. (67)	USA	Cross-sectional	Medical records	n.m.	66.9
Kaess et al. (68)	Australia	Cross-sectional	Orygen adolescent development study	n.m.	19.0 (CTQ > 35)
Malhi et al. (69)	Australia	Cross-sectional	Community	n.m.	37.8
Östberg et al. (70)	Sweden	Cross-sectional	School stress and support study	2010	13.5
Pagliaccio et al. (71)	USA	Cross-sectional	Preschool depression study	n.m.	n.m.
Ruttle et al. (72)	USA	Longitudinal	Wisconsin study of families and work	n.m.	n.m.
Saxbe et al. (73)	USA	Longitudinal	Urban sample	n.m.	n.m.
			Longitudinal study of youth		
Simsek et al. (74)	Turkey	Case-control	Department of Child Psychiatry at Dicle University Hospital	December 2011 and April 2012	n.m.
**Mid adolescence: 13–15 years**					
Efstathopoulos et al. (75)	Sweden	Cross-sectional	KUPOL project	2013–2014	n.m.
				2014–2015	
**Late adolescence: 15–18 years**					
Edmiston et al. (76)	USA	Cross-sectional	Community	n.m.	85.7
Esposito et al. (77)	USA	Case-control	Community	n.m.	n.m.
**0–18 years**					
Marzi et al. (78)	United Kingdom	Longitudinal	Environmental risk longitudinal study	1999–2000; 2001–2002; 2006–2007; 2012–2013	28.1
Radtke et al. (79)	Germany	Cross-sectional	Community	n.m.	n.m.
Serbulent et al. (80)	Turkey	Case-control	Department of child protective service	May 2016–July 2016	Amongst cases: 63.0
Tyborowska et al. (81)	Netherlands	Longitudinal	Nijmegen longitudinal study on child and infant development	n.m.	n.m.
Van Der Knaap et al. (82)	Netherlands	Longitudinal	Tracking adolescents' individual lives survey	2001–2002 2003–2004 2005–2007 2008–2010	Physical abuse: 38.7 Sexual abuse: 7.1 Other trauma: 24.8
van der Knaap et al. (83)	Netherlands	Longitudinal	Tracking adolescents' individual lives survey	2001–2002 2003–2004 2005–2007 2008–2010	Physical abuse: 35.5 Sexual abuse: 7.0 Other trauma: 22.6
White et al. (84)	Germany	Case-control	Community	n.m.	n.m.

**Table 4:** Direction and strength of association between exposure to ACEs and biomarker by biological mechanism (positive associations indicate that biomarker increases with ACEs exposure and/or frequency; inverse associations indicate that biomarker decreases with ACEs exposure and/or frequency).

**References**	**Biomarker**	**Type of ACEs**		**Direction of association**	**Strength of association**
**Genetic and epigenetic**					
Bush et al. (43)	DNA methylation	Life stressors^*^		Positive	Weak to moderate
Cicchetti et al. (63)		Abuse		Positive	Strong
Cicchetti et al. (44)		Abuse and neglect		Positive	Strong
Fujisawa et al. (32)		Physical, emotional and sexual abuse, physical, and emotional neglect		Positive	Strong
Naumova et al. (52)		Foster care		Positive	Strong
Non et al. (53)		Foster care		Inverse	Strong
Parade et al. (36)		Physical and sexual abuse, physical neglect, and emotional maltreatment		Inverse	Strong
Parent et al. (37)		Physical and sexual abuse, physical neglect, and emotional maltreatment		Positive	Strong
Tyrka et al. (38)		Physical and sexual abuse, physical neglect, and emotional maltreatment		Positive	Strong
Van Der Knaap et al. (82)	NR3C1 CpG1	Life stressors^*^		Positive	Strong
	NR3C1 CpG2			Positive	Strong
	NR3C1 CpG3			Inverse	Strong
Yang et al. (62)	DNA methylation	Physical, sexual, emotional abuse, and witnessed domestic violence		Positive	Strong
Esposito et al. (77)		Life stressors^*^		Positive	Weak
Van Der Knaap et al. (83)	SLC6A4	Life stressors^*^		Positive	Strong
Chen et al. (29)	ADCYAP1R1	Life stressors^*^		Positive	Weak
Cicchetti and Handley (30)	NR3C1	Abuse and neglect		Positive	Strong
Marzi et al. (78)	NR3C1	Domestic violence, bullying, physical maltreatment, sexual abuse, emotional abuse and neglect, and physical neglect		Positive	Weak
Radtke et al. (79)	NR3C1	Life stressors^*^, physical, emotional and sexual abuse, physical and emotional neglect		Positive	Strong
Romens et al. (55)	NR3C1	Physical maltreatment		Positive	Strong
Tyrka et al. (39)	FKBP5	Physical and sexual abuse, physical neglect and emotional maltreatment	Adversity composite	Inverse	Strong
			Lifetime contextual stress	Positive	Strong
			Past-month contextual stress and the number of traumatic life events	No association	-
Efstathopoulos et al. (75)	NR3C1	Bullying and life stressors^*^		Positive	Strong
Shalev et al. (33)	Telomere length	Life stressors^*^, bullying and physical maltreatment		Inverse	Strong
Drury et al. (50)		Life stressors^*^		Inverse	Strong
**Immune system**					
Bevans et al. (41)	Cortisol	Life stressors^*^ (within the past 12 months, recent and frequent trauma and afternoon cortisol)		Positive	Strong
		Life stressors^*^ (within the past 12 months, recent and frequent trauma and morning cortisol)		No association	-
Bhopal et al. (26)	Cortisol	Life stressors^*^		Positive	Strong
Bruce et al. (35)		Physical and sexual abuse, physical neglect, and emotional maltreatment		Positive	Strong
		Physical neglect (severity)		Inverse	Strong
Buchweitz et al. (42)		Life stressors^*^		Positive	Strong
Cicchetti et al. (45)		Abuse and neglect		Positive	Strong
Doom et al. (48)		Physical, emotional and sexual abuse, physical, and emotional neglect		Positive	Strong
Doom et al. (49)		Abuse and neglect		Positive	Strong
Östberg et al. (70)		Bullying (girls)		Inverse	Weak
		Bullying (boys)		Inverse	Strong
Ruttle et al. (72)		Life stressors^*^		No association	-
Simsek et al. (56)		Sexual abuse		Positive	Strong
Simsek et al. (74)		Sexual abuse		Positive	Strong
Stroud et al. (57)		Life stressors^*^		Inverse	Strong
Trickett et al. (58)		Sexual abuse		Inverse	Strong
Vaillancourt et al. (59)		Bullying		No association	-
White et al. (84)		Physical and sexual abuse, physical neglect, and emotional maltreatment		Inverse	Strong
Serbulent et al. (80)		Sexual abuse		No association	-
	IL-6			Positive	Strong
	IL-10			No association	-
Baldwin et al. (40)	CRP	Several types of victimization (girls)		Positive	Strong
Cicchetti et al. (31)		Abuse and neglect (only for those with at least one A allele)		Positive	Weak
Copeland et al. (65)		Bullying		No association	-
		Bullying (victims)		Positive	Strong
		Bullying (bullies)		Inverse	Strong
		Bullying (bully-victims)		No association	-
Danese et al. (47)		Physical maltreatment		No association	-
Bucker et al. (28)	IL-12p70	Sexual abuse, maltreatment, and/or neglect		No association	-
	IL-6			No association	-
	IL-8			No association	-
	IL-10			No association	-
	IL1β			No association	-
	TNF-α			Positive	Strong
	BDNF			Positive	Strong
Slopen et al. (34)	IL-6	Life stressors^*^ and sexual abuse		Positive	Strong
	CRP			Positive	Strong
**Structural and functional brain changes**					
Cisler (64)	Amygdala functional connectivity	Physical, emotional and sexual abuse, physical and emotional neglect		Inverse	Strong
Pagliaccio et al. (71)		Life stressors^*^		Positive	Strong
Park et al. (54)		Life stressors^*^		Inverse	Strong
Dahmen et al. (27)	Hippocampal volume	Maltreatment		Inverse	Strong
Edmiston et al. (76)	Gray matter	Physical, emotional and sexual abuse, physical, and emotional neglect		Inverse	Strong
Tyborowska et al. (81)		Life stressors^*^		Inverse	Strong
Humphreys et al. (66)	Hippocampal volume	Life stressors^*^, physical, and sexual abuse		Inverse	Moderate
Kaess et al. (68)	Pituitary gland volume	Physical, emotional and sexual abuse, physical, and emotional neglect		Positive	Weak
Whittle et al. (61)	Hippocampal volume	Physical and sexual abuse, physical neglect, and emotional maltreatment		Positive	Strong
	Amygdala volume			Inverse	Strong
Malhi et al. (69)	Hippocampal volume	Emotional abuse and/or neglect		Inverse	Strong
Saxbe et al. (73)	Hippocampal volume	Life stressors^*^		Inverse	Strong
	Amygdala volume			Inverse	Weak
Simsek et al. (74)	BDNF	Sexual abuse		Inverse	Strong
Ito et al. (67)	Neurological abnormalities	Physical, emotional, and sexual abuse		No association	-
Huang et al. (51)	Voxel-based morphometry	Physical abuse, sexual abuse, and/or witnessed domestic violence		Inverse	Strong
**Other**					
Coelho et al. (46)	Copeptin	Physical, emotional, sexual abuse, physical, and emotional neglect		Positive	Strong
Doom et al. (49)	DHEA	Abuse and neglect (boys)		Inverse	Strong
Danese et al. (47)	Leptin	Physical maltreatment		Positive	Strong

* *Life stressors (e.g., death of a family member, trouble with a teacher)*.

### The Methodological Quality of Studies

The quality of reporting of the included studies was assessed using the Strengthening Reporting of Observational Studies in Epidemiology (STROBE) Statement: Guidelines for Reporting Observational Studies (86). All studies scoring higher than the median in the STROBE checklist for cohort, case-control, and cross-sectional studies (combined) and thus revealing a satisfactory to good quality were included (Table 2).

## Results

The characteristics of the 58 included publications are described in Tables 2, 3.

Twelve studies were conducted in Europe (5 countries), 36 in the Americas (4 countries), six in Asia (4 countries), three in Australia and one in Africa (Tanzania). Most studies were conducted in the United States of America (USA) (31 studies), and the sample size varied from 21 to 5,802 participants. Studies were divided according to the time at which ACEs occurred. The distribution of papers is as follows: two papers during toddlerhood, 34 studies during childhood (seven from 3 to 12 years, five from play - 3 to 5 years- and 21 from middle childhood − 6–12 years), 15 studies during adolescence (12 studies in adolescence−13–18 years, one in mid-adolescence−13–15 years and two in late adolescence−15–18 years) and seven studies that present ACEs measured from an overall period—comprising experiences occurred before 18 years (Tables 2, 3). In childhood, most publications (15 studies) are in the “immune system” and “genetic and epigenetic” categories, while “structural and functional brain changes” has three publications. During adolescence there are six publications with biomarkers from the “immune system,” nine studies from the “structural and functional brain changes,” and seven studies from the “genetic and epigenetic” category.

**Figure 2:**
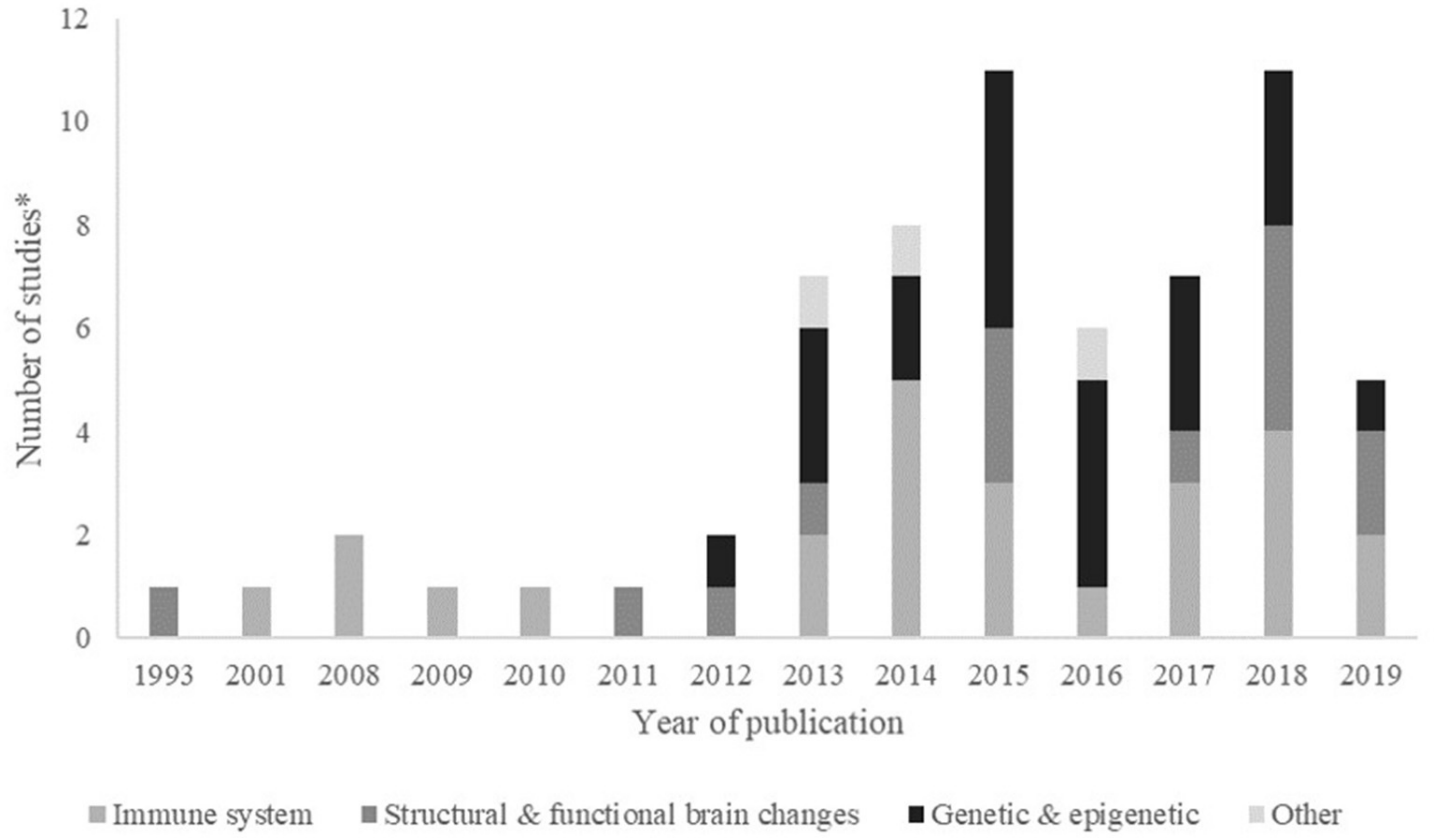
Frequency (number of studies*) by biological mechanism, published per year. *Some papers evaluated more than one biomarker, within or not the same biological mechanism. Year 2019 includes papers published online until July 2019.

Publication of studies increased over time, with most of the studies being published after 2012. The first study using DNA methylation as a biomarker of exposure to adversity was published in 2012, and after that, the number of papers studying the association with genetic and epigenetic biomarkers has been consistently increasing (Figure 2).

We observed that ACEs were mostly assessed by standardized instruments, although some authors used non-validated questions (17 studies). The most frequently used instrument was the Childhood Trauma Questionnaire (nine studies), followed by the Maltreatment Classification System (eight studies). High heterogeneity was found among studies both in the exposure measurement and in the outcome summary measures. Regarding exposure, the most frequent adverse event measured in these studies was sexual abuse (26 studies, 16 studies in childhood and 10 studies in adolescence), followed by the life stressors category, that includes the death of a family member, trouble with a teacher, exposure to community violence, among others (20 studies, 10 in childhood, and 10 in adolescence), by physical abuse (18 studies, 11 studies in childhood and seven in adolescence) and physical neglect (15 studies, nine studies in childhood and six in adolescence). The minimum time from exposure to ACEs and measurement of biomarkers was 72 h and a maximum of 18 years. In toddlerhood the average time between exposure to ACEs and measurement of biomarkers was 2 years, in childhood was 7.2 years and during adolescence was 5.5 years (Table 2).

We categorized papers according to the outcome measured, i.e., referring to the biological marker used to assess the effect of ACEs on biological mechanisms. Biological markers were then divided by the biological mechanism with which they fitted better: “immune system” (including CRP, IL-6, TNF-α, IL-1b, IL-10, IL-12p70, IL-8, and cortisol), “structural and functional brain changes” (BDNF, hippocampal volume, amygdala volume, amygdala functional connectivity, gray matter, neurologic abnormalities, pituitary gland volume, voxel-based morphometry), “genetic and epigenetic” (including methylation and telomere length) and others [including copeptin, leptin, and dehydroepiandrosterone (DHEA)].

In almost all studies, exposure to ACEs was associated with biomarker alterations already during childhood, while six found no evidence of effect modification (Table 4).

Mainly due to the nature and type of biomarkers, associations observed can be expressed through an increase or a decrease in respective biomarkers. An increase is mainly reflected if higher biomarker levels are observed after exposure to ACEs than it would be expected if no exposure to ACEs occurred. A decrease will be defined if the observed biomarker levels are lower than after exposure to ACEs than they would be if no exposure to ACEs were in place. Thirty-nine studies presented a positive association, meaning that participants exposed to adverse experiences presented higher levels of biological markers, and 29 studies showed inverse associations, corresponding to a decrease in the biological markers when adversity was reported. We observed that most authors study the association of ACEs with biomarkers of the immune system followed by genetic and epigenetic biomarkers and then structural and functional brain changes.

### Biomarkers

#### Immune System

**Figure 3:**
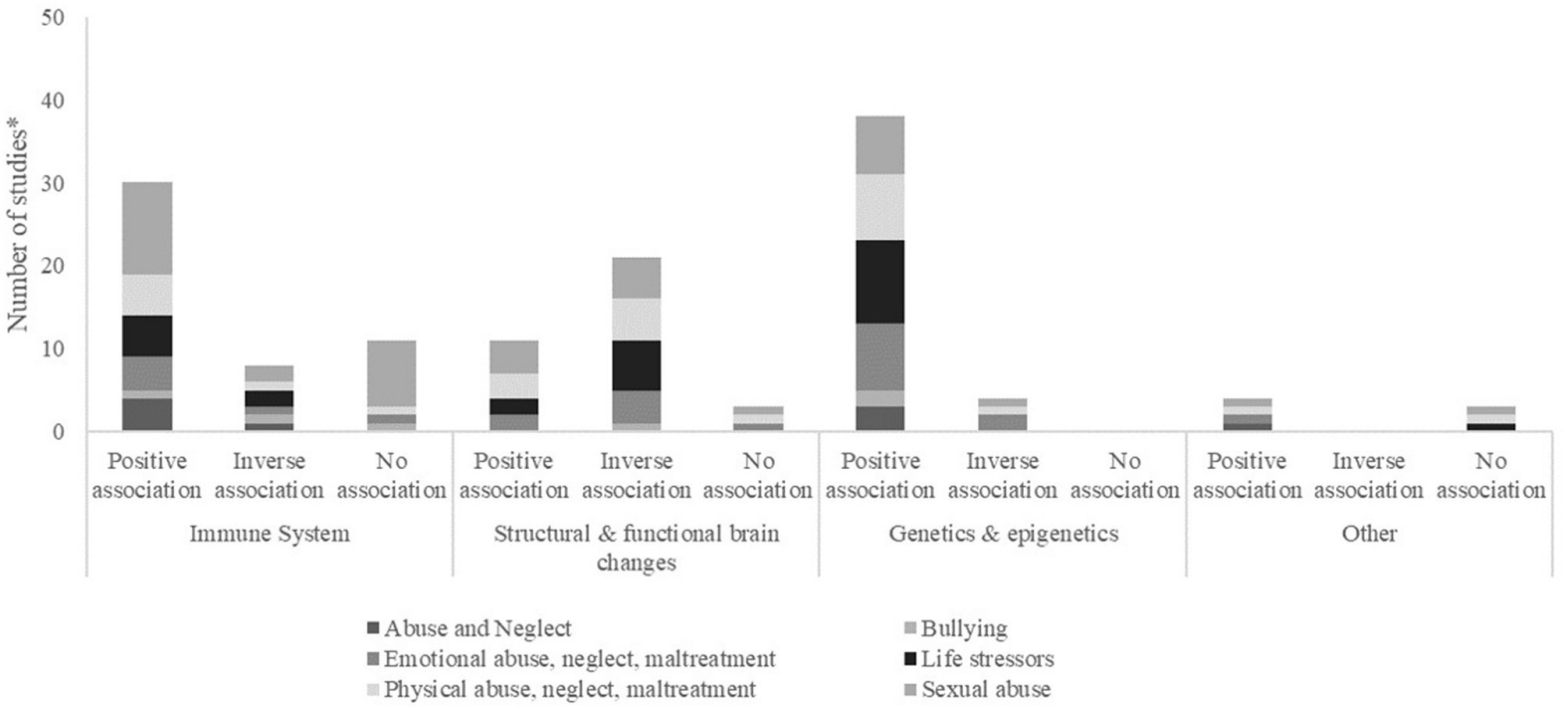
Frequency (number of studies*) categorized by presence of association (positive, negative) or absence of association (no association) found between exposure to ACEs and biomarker, by biological mechanism and type of ACE. *Some papers evaluated more than one biomarker, within or not the same biological mechanism.

Of the studies that addressed the biological consequences of ACEs on the immune system, 16 focused on cortisol, five on CRP, three on IL-6, two on IL-10, one on TNF-α, IL-1b, IL-12p70 and IL-8. Of these, the majority (17 studies) showed a positive association between ACEs exposure and biomarkers of the immune system, meaning that those exposed to adverse experiences presented higher levels of biomarkers of the immune system. Other studies showed an inverse association (five studies), with exposure to ACEs being associated with lower levels of biomarkers, or no association (five studies). The majority of studies presented strong associations, while four publications reported weak associations between exposure to ACEs and biomarkers. Regarding the type of ACEs more associated with biomarkers of the immune system, we saw that the categories sexual abuse, life stressors and physical abuse, neglect, maltreatment were the more prevalent (Figure 3). Changes in cortisol levels can be observed as early as between 3 and 6 years. Also, analyzing the distribution of publications by age, 11 studies on the immune system were conducted between the ages of 6 and 12 years. Four studies were conducted between 13 and 18 years and three between 3 and 12 years.

#### Structural and Functional Brain Changes

The authors measured the impact of ACEs in the structural and functional brain changes, using several types of outcomes. Hippocampal volume was measured in five studies, and amygdala functional connectivity in three, BDNF, amygdala volume and gray matter in two, neurologic abnormalities, pituitary gland volume and voxel-based morphometry in one study each. Of these, three studies showed a moderate or weak association between exposure to ACEs and the outcomes measured, while all the others presented a strong association. Most studies showed an inverse association of ACEs, namely when reporting the association between sexual abuse, life stressors and physical abuse, neglect, maltreatment with structural and functional brain changes (Figure 3). Amygdala functional connectivity was the biomarker of the group “Structural and functional brain changes,” that presented changes measured earlier (mean = 6.1 years). Examining the distribution of publications by age, the majority of studies in this category (seven studies) were conducted between the ages of 13 and 18 years, and three studies were conducted between 6 and 12 years.

#### Genetic and Epigenetic

DNA methylation was assessed in 20 studies, with 18 showing more methylation in participants exposed to ACEs, four showing less methylation and one reporting no association. Methylation is observed in a multiplicity of genes or focused on specific genes, such as NR3C1, SLC6A4, and FKBP5. The effect of ACEs on telomere length was presented in two studies and showed that exposure was associated with shorter telomere length. The majority of associations observed was regarding the association with sexual abuse, life stressors and physical abuse (Table 4). DNA methylation is altered as early as between 3 and 5 years, and changes in telomere length can be observed at 10.2 years. Also, analyzing the distribution of publications by age, eight studies of the “genetic and epigenetic” category were conducted between 3 and 12 years and seven studies were conducted between the ages of 6 and 12 years.

#### Others

One study evaluated copeptin, and other DHEA, and both showed a positive association with ACEs exposure and were conducted in middle childhood, i.e., between 6 and 12 years old. A study on leptin showed no association with ACEs.

### Exposures

#### Types of Abuse

Among the 11 studies evaluating abuse (28, 30, 38, 39, 44, 45, 48, 49, 62, 82, 83), only three present the associations with biomarkers stratified by types of abuse (45, 82, 83). In one study (45), it was observed that average morning cortisol in the group of sexually and physically abused participants (*M* = 0.99, SD = 0.83) was higher than in the non-maltreated (*M* = 0.32, SD = 0.67), emotionally maltreated (*M* = 0.35, SD = 0.58), neglected (*M* = 0.28, SD = 0.65), and physically abused (*M* = 0.14, SD = 0.62) participants. Regarding methylation results, results of linear regression models (82) showed that exposure to stressful life events between birth and 15 years and exposure to traumatic youth experiences significantly predicted higher methylation rates in amplicon 1. In amplicon 2, an only single exposure to sexual abuse predicted higher methylation rates (*B* = 0.44, *P* = 0.001). For amplicon 3, repeated exposure to other traumatic youth experiences was associated with lower methylation rates (*B* = −0.26, *P* = 0.01). The other study (83) reports that exposure to perinatal adversity or traumatic young experiences was not related to methylation, while exposure to stressful life events in the first 15 years of life significantly predicted higher methylation levels. In the model including both stressful life events during childhood and adolescence, exposure to stressful life events in adolescence was related to higher methylation levels.

#### Bullying Involvement

Six studies were identified evaluating bullying as an experience of adversity. One studied the impact of bullying on CRP, and found that CRP levels were higher in victims of bullying and lower in aggressors (65); two studies reported that DNA methylation was higher among victims of bullying (75, 78), and other that telomere length was shorter (33). Two other studies evaluated cortisol and one described lower levels of cortisol among victims of bullying (70) while another study found no association (59).

## Discussion

This review shows that exposure to ACEs might impact the immune system, structural and functional brain changes and genetic and epigenetic changes, and these changes can be observed as early as childhood. However, a high heterogeneity is observed between included studies in ACEs measures, analytic methods and heterogeneity in the biomarkers.

### Assessment of ACEs Among Children

In these studies, ACEs were assessed through different methods of inquiry and instruments. The development and testing of measures of retrospective adult recall of ACEs have been a fruitful area of research for the past few decades with several measures being developed and field-tested. Thus, most studies used retrospective measures to identify exposure to ACEs. The major issue raised is that several critical aspects of the measurement systems are inconsistent across studies, making it difficult to synthesize knowledge generated to date (87). In this review, by focusing on studies that assess exposure and outcome measured in the first 18 years of life, we see that biological alterations caused by exposure to traumatic events can be observed in the first years of life. The majority of included publications studies the effect of adversity in toddlerhood and childhood, i.e., before the age of 12 years (36 studies) while 15 studies evaluated adverse experiences between 13 and 18 years of age.

The heterogeneity on measurement instruments used gives rise to another assessment inconsistency, in particular, the fact that not all types of victimization are alike. The majority of studies presents results by adversity composite or number of adversities, by chronicity or timing of abuse, and few have analyzed by type of trauma. Some involve physical injury (sexual or physical abuse), whereas others involve psychological insult (emotional abuse or neglect). Also, some papers refer only to one type of adversity while others report several exposures to ACEs. In this review, we observed that sexual abuse was, among the categories of ACEs studied, the type of adversity that most studies presented in association with different biomarkers. This might be explained by the fact that the biological embedding of social experiences occurs sooner when the experience is very traumatic or repeated over time (88).

### Potential Biological Mechanisms to the Embodiment of ACEs

The impact of ACEs in the immune system, structural and functional brain, as well as the genetic and epigenetic changes, was explored in the reviewed studies including samples of children. Overall, the associations observed followed the hypothesis that ACEs are associated with biological risk, which can be expressed through increases or decreases in respective biomarker levels above or under the expected levels if no exposure to ACEs was in place, depending on the nature and type of biomarker.

Immune and non-immune cells produce cytokines, messenger proteins such as TNF-α, IL-1β, IL-8, IL-6, IL-10, and IL-12p70, whose role is to regulate immune responses and interplay between pro and anti-inflammatory mediators (89, 90). CRP is an acute-phase protein synthesized by the liver in response to systemic effects of inflammation (91) and may intervene in the biological chain that embeds exposure to ACEs. Cortisol is the product of the HPA axis and has been widely used as a stress biomarker. All of these biomarkers play a role in the regulation of the immune responses and interplay between pro and anti-inflammatory mediators (89, 90) indicating an interrelated activation of the entire inflammatory cascade (92). More recently, evidence has reviewed the effect of early exposure to adversity on the chronic inflammatory state (23, 93) and concluded that early adversity is likely to increase inflammation (18, 23, 24, 93) and risk for poor health outcomes in adulthood (8, 93), independent of clinical comorbidities (23, 24). Our results show that these biomarkers seem to present alterations in the first 18 years of life, and thus the effect of exposure to childhood adversity in the immune system, in particular in the inflammatory biomarkers, where alterations were reported as early as between 3 and 6 years.

Several papers included in this review assessed methylation in a multiplicity of genes or focused on specific genes, such as NR3C1, SLC6A4, and FKBP5. These three genes seem to play an active role in the biological embodiment of exposure to ACEs and we hypothesized that the effect of adversities would be observed on alterations already at early ages. On one hand, NR3C1 is a gene known to encode glucocorticoid receptor, involved in inflammatory responses (94), and the higher level of methylation has been associated with childhood violence (95); and the SLC6A4 gene that encodes an integral membrane protein and seems to play a role in depression-susceptibility in people experiencing emotional trauma (96). FKBP5 encodes to a protein member of the immunophilin protein family, which play a role in immunoregulation and basic cellular processes. Genetic studies have identified a role of this gene in post-traumatic stress disorder, depression and anxiety (97) and have been found to interact with childhood trauma to predict the severity of adult post-traumatic stress disorder (98).

Although multiple types of epigenetic modifications have already been identified (99), all involve chemical modifications that regulate chromatin structure and/or DNA accessibility. Methylation, corresponding to the covalent modification of DNA whereby methyl groups are coupled to cytosine residues at CpG sites, is perhaps the best studied of these epigenetic mechanisms, due in part to its tractability (100). In this review, we identified several studies evaluating DNA methylation after exposure to ACEs. As dynamic molecular markers that have been shown to change with age (101) and experience (102), epigenetic signatures are attractive candidates for elucidating the underlying mechanisms of complex diseases (103).

Emerging evidence shows that environmental signals give rise to epigenetic changes, affecting phenotypic trajectories by altering the expression of genes (104). Thus, changes in epigenetic regulation of gene expression seem to be responsible for an increased immune activation *via* modifications of the HPA axis. Neuroplasticity-related methylation patterns (13, 105) may be a possible mechanism through which the association between early adverse experiences and long-term alterations in human stress response and immune systems are mediated.

Also, although not very conclusive, some structural and functional brain changes after exposure to adverse experiences have been identified by the studies explored in the review. Six studies concluded that hippocampal and amygdala volume and gray matter decreased after participants experienced adverse experiences. However, more evidence is needed to have a comprehensive view of the effect of ACEs in these systems.

### Impact of ACEs on the Physiological Systems

Most of the included studies showed a significant impact of ACEs on the different physiological systems. Nevertheless, some studies showed increases in biomarker levels, while others presented decreases in those levels, depending on the nature and type of biomarker. Regarding telomere length, amygdala and hippocampal volume, the direction of the observed associations was consistent with our hypotheses. Telomere length decline is a normal consequence of cellular division, aging, differentiation, and senescence. Accelerated telomere shortening in adults has been associated with a history of childhood maltreatment and early adversity (106, 107). DNA methylation also can occur *via* hypermethylation, i.e., increased methylation, that was found in the promoter region of SLC6A4 in adult men after early and recent life stress (108), or hypomethylation, i.e., decreasing methylation, observed at intron 7 of FKBP5 in adults exposed to childhood trauma (109). Thus, the direction of methylation may depend on the gene, promoter and/or region studied. However, we did not expect to find different directions of association for biomarkers such as cortisol. But, there is some evidence of the attenuation hypothesis (110), suggesting that exposure to early and severe stress leads to an initial heightened stress response, that may be suppressed over time. This suppression may be suggestive of an adaptive response. Cortisol levels increase immediately after exposure to ACEs, and attenuate after a certain time, but continue to reflect the effects of severe trauma. Evidence from primates showed that early life stressors, when not tremendously severe, were associated with the subsequent development of biological and social resilience suggesting that ACEs represent a challenge that, when overcome, bring about functional adaptations (111). Regarding amygdala functional connectivity, some inconsistencies might be explained by within-subject variability and fluctuations in large-scale network patterns, including connectivity between a limbic and default mode network, results that seem to suggest that bi-nodal functional connectivity, may generally reflect larger-scale network patterns.

Additionally, our review shows that age at exposure is very different across publications, varying from <6 months to under 18 years old. The wide range of ages included is due to the inclusion of all experiences occurred before adult life, and thus during the major period of growth and development of a human being. Although there is great variability across studies, it has been defended that given the vast array of developmental processes occurring between conception and adolescence, every developmental window is in fact characterized by a different susceptibility depending on various environmental factors (112).

With this review, we cannot assess if the experiences reported are single episodes or if they are related to several experiences throughout childhood and adolescence resulting in cumulative exposures during these maturation periods. The exception is one study that specifically states that adversity must last for at least 6 months (51). There is evidence showing that cumulative exposures seem to have stronger associations with later health outcomes (1). This means that we could be looking at an interplay between biological functions and the environment across the life course which we cannot disentangle from the mechanism of accumulation. For example, an individual most at risk of developing cancer or ischemic heart disease after childhood exposure to violence or adversity is also more likely to have accumulated further negative experiences over time and to adopt risky health behaviors as a stress-reducing escape. However, by restricting the search to studies with participants 18-year-old or younger, the time for accumulation of risk-taking behaviors is sufficiently limited to avoid an impact on the studied association. Moreover, when compared to adult life, neurodevelopment during childhood and adolescence is more plastic and susceptible to programming influences from stressful environmental and social contexts (113). Also, even though there is evidence that different biomarkers show alterations upon exposure to ACEs, we cannot disregard that these alterations do not necessarily mean an increased risk of disease onset. The development and progression of disease may occur due to the interplay of a group of correlated molecules or a network, rather than from the malfunction of the individual gene, protein, or biomarker.

### Biological Consequences of Bullying Involvement

It is not consensual to include bullying as an adverse experience in childhood. However, the awareness of this problem has widely increased, and it was shown to compromise the child's health. Literature settles on the conviction that social and psychological effects of bullying involvement may be independent of other childhood experiences (114), but the biological mechanisms of the embodiment of these experiences are still not fully elucidated. Although some authors agree that one potential mechanism is related to the chronic systemic low-grade inflammation (115), once inflammation is activated similarly by a diverse range of health risky behaviors (poor diet, sedentary life) and environmental challenges (low socioeconomic status, psychosocial stress) (116), others support the hypothesis of embodiment throughout HPA axis activation or autonomic nervous system (ANS) activation. Bullying has also some specificity as the type of involvement, as the victim or as the aggressor or both simultaneously might have a different biological impact. Evidence has shown that although being bullied predicted higher increases in CRP levels, bullying others predicted lower increases in CRP compared with those uninvolved in bullying, even when controlling for potential confounders (65). This review identified six studies evaluating bullying as an experience of adversity. Thus, further investigation is needed to explore the impact of children's type of involvement in bullying on different biological markers.

Nowadays, another important and prevalent form of bullying is by using technologies and social media, named cyberbullying. Due to the potential of widespread accessibility of victims and an infinite audience by using communication technologies (117), cyberbullying is another important source of stress and consequently to biological alterations that can later lead to disease. This is another important issue that deserves attention in future studies.

### Strengths and Limitations

We believe that this review is comprehensive and robust enough to show the studied association. Even though there is always the possibility of residual confounding when exploring the association between childhood exposure and biological markers, we believe that studying these biomarkers already during childhood is an important step to eliminate the effect of health-risk behaviors that may confound this association. We must acknowledge that different biological, psychological and social aspects may contribute to the changes in the biomarkers studied, which are difficult to control for. However, our results are in line with previously reported associations (23, 104, 118, 119), and allow us to retrieve important conclusions on the effect of early exposure to ACEs and alterations in human stress response and biological systems, already during childhood. The reported biomarkers were also chosen based on previously published literature, and others emerged from the search, showing that several systems may be affected by adverse experiences in childhood. Even though we cannot exclude the hypothesis that more biomarkers might be affected by these experiences, we believe that our comprehensive search allowed us to catch most studies. Nevertheless, excluding allostatic load (AL) from our search might be considered a limitation. AL is posited to represent a sub-clinical measure of physiological wear and tear resulting from chronic exposure to life course stressors providing a measure of cumulative physiological dysregulation across multiple biological systems. We did not include the AL in our search because, despite its apparent utility, AL is affected by many methodological and conceptual choices that have hampered its potential clinical utility. Among those, we may highlight the difficulty to agree on a core set of biomarkers that define the construct, and the different AL scoring algorithms, limiting our ability to compare results across studies. Moreover, the cumulative nature of the allostatic load, identifying which biological system would suffer the most the impact of exposure to adversity would be more difficult. Also, to our knowledge, only one publication assessed the effect of maltreatment on allostatic load in children (120). In this study (120), participants were aged 8–10 years, included maltreated or non-maltreated low-income children that attended a summer research day camp. The authors observed that maltreatment did not independently predict differences in allostatic load levels.

Additionally, due to the diversity of ACEs measures, analytic methods and heterogeneity in the biomarkers we were not able to calculate a summary measure of association between ACEs and biological markers, and thus we were unable to conduct a meta-analysis. Instead, a qualitative description of the strength of association was assigned based on the magnitude of effect measures.

None of the exclusion criteria chosen to conduct this review is related to any aspect of human differences such as socioeconomic status, race, ethnicity, language, nationality, sex, gender identity, sexual orientation, religion, geography, ability, age, or culture. Thus, this review holds diversity as a core value and all papers were included based on the criteria defined and no other.

Understanding the biological mechanisms between ACEs and negative health outcomes is important as it offers avenues for treatments that could target these intermediary pathways to prevent or reduce the risk and burden of diseases such as cancer and cardiovascular disease. Nevertheless, we must be aware that some of the associations may be mediated by depression (121) or even life course socioeconomic and health behavioral factors (18), as these factors have been suggested as impacting inflammatory processes.

## Conclusion

Despite the considerable inconsistency in ACEs assessment, most articles reviewed found an association between exposure to ACEs and biological markers, where the increase or decrease in the biomarker is associated with a heightened risk to subsequent health. Experiences of violence in childhood appear to “get under the skin” and induce physiological changes, such as increases in immune, structural, and functional brain changes, and genetic and epigenetic markers, from childhood. Thus, supporting evidence of a more immediate biological impact of these exposures and alterations might be strongly associated with the later development of disease. These results allow us to argue that the population's burden of disease could be reduced if all violence toward children was successfully prevented (122) and when it does occur, appropriately treated to mitigate the consequences (123). Exposure to adverse childhood experiences should be prevented as a question of human rights, and children should be protected against all types of abuse by law enforcement and providing nurturing childhood environments. Moreover, as adverse experiences seem to impact children's biology and children may be growing in a trajectory of worse health throughout life, beginning at early ages, when exposure to adversity cannot be prevented, clinicians may have an important role in helping identify any biological alterations related with adversity victimization and intervene to mitigate their impact on health.

## Data Availability Statement

The original contributions presented in the study are included in the article/supplementary material, further inquiries can be directed to the corresponding author.

## Author Contributions

SSo and SF designed the study and wrote the protocol. SSo managed the literature searches, extraction of data and analyses, and wrote the first draft of the manuscript. VR collaborated in the extraction of data. SF helped solve differences in the data extraction. SF, MK-I, and SSt reviewed and discussed the manuscript. All authors contributed to and have approved the final manuscript.

## Conflict of Interest

The authors declare that the research was conducted in the absence of any commercial or financial relationships that could be construed as a potential conflict of interest.

## Publisher's Note

All claims expressed in this article are solely those of the authors and do not necessarily represent those of their affiliated organizations, or those of the publisher, the editors and the reviewers. Any product that may be evaluated in this article, or claim that may be made by its manufacturer, is not guaranteed or endorsed by the publisher.
